# Psychosocial Outcomes Among Users and Nonusers of Open-Source Automated Insulin Delivery Systems: Multinational Survey of Adults With Type 1 Diabetes

**DOI:** 10.2196/44002

**Published:** 2023-12-14

**Authors:** Jasmine Schipp, Christel Hendrieckx, Katarina Braune, Christine Knoll, Shane O’Donnell, Hanne Ballhausen, Bryan Cleal, Mandy Wäldchen, Dana M Lewis, Katarzyna A Gajewska, Timothy C Skinner, Jane Speight

**Affiliations:** 1 The Australian Centre for Behavioural Research in Diabetes Carlton Australia; 2 Section for Health Services Research, Institute of Public Health University of Copenhagen Copenhagen Denmark; 3 School of Psychology Deakin University Burwood Australia; 4 Department of Paediatric Endocrinology and Diabetes Charité – Universitätsmedizin Berlin Berlin Germany; 5 Institute of Medical Informatics Charité – Universitätsmedizin Berlin Berlin Germany; 6 Berlin Institute of Health Berlin Germany; 7 Dedoc Labs GmbH Berlin Germany; 8 School of Sociology & School of Medicine University College Dublin Dublin Ireland; 9 Diabetes Management Research Steno Diabetes Center Copenhagen Copenhagen Denmark; 10 #OpenAPS Seattle, WA United States; 11 Diabetes Ireland Dublin Ireland; 12 School of Public Health University College Cork Cork Ireland

**Keywords:** artificial, diabetes mellitus, hypoglycaemia, pancreas, patient-reported outcome measures, surveys, and questionnaires, quality of life, sleep quality, type 1

## Abstract

**Background:**

Emerging research suggests that open-source automated insulin delivery (AID) may reduce diabetes burden and improve sleep quality and quality of life (QoL). However, the evidence is mostly qualitative or uses unvalidated, study-specific, single items. Validated person-reported outcome measures (PROMs) have demonstrated the benefits of other diabetes technologies. The relative lack of research investigating open-source AID using PROMs has been considered a missed opportunity.

**Objective:**

This study aimed to examine the psychosocial outcomes of adults with type 1 diabetes using and not using open-source AID systems using a comprehensive set of validated PROMs in a real-world, multinational, cross-sectional study.

**Methods:**

Adults with type 1 diabetes completed 8 validated measures of general emotional well-being (5-item World Health Organization Well-Being Index), sleep quality (Pittsburgh Sleep Quality Index), diabetes-specific QoL (modified DAWN Impact of Diabetes Profile), diabetes-specific positive well-being (4-item subscale of the 28-item Well-Being Questionnaire), diabetes treatment satisfaction (Diabetes Treatment Satisfaction Questionnaire), diabetes distress (20-item Problem Areas in Diabetes scale), fear of hypoglycemia (short form of the Hypoglycemia Fear Survey II), and a measure of the impact of COVID-19 on QoL. Independent groups 2-tailed *t* tests and Mann-Whitney *U* tests compared PROM scores between adults with type 1 diabetes using and not using open-source AID. An analysis of covariance was used to adjust for potentially confounding variables, including all sociodemographic and clinical characteristics that differed by use of open-source AID.

**Results:**

In total, 592 participants were eligible (attempting at least 1 questionnaire), including 451 using open-source AID (mean age 43, SD 13 years; n=189, 41.9% women) and 141 nonusers (mean age 40, SD 13 years; n=90, 63.8% women). Adults using open-source AID reported significantly better general emotional well-being and subjective sleep quality, as well as better diabetes-specific QoL, positive well-being, and treatment satisfaction. They also reported significantly less diabetes distress, fear of hypoglycemia, and perceived less impact of the COVID-19 pandemic on their QoL. All were medium-to-large effects (Cohen *d*=0.5-1.5). The differences between groups remained significant after adjusting for sociodemographic and clinical characteristics.

**Conclusions:**

Adults with type 1 diabetes using open-source AID report significantly better psychosocial outcomes than those not using these systems, after adjusting for sociodemographic and clinical characteristics. Using validated, quantitative measures, this real-world study corroborates the beneficial psychosocial outcomes described previously in qualitative studies or using unvalidated study-specific items.

## Introduction

Type 1 diabetes is a chronic auto-immune condition caused when the pancreas stops making the hormone insulin, which is essential to regulating blood glucose levels. Facing the daunting task of trying to imitate a functioning pancreas, people with type 1 diabetes must perform complex and relentless self-care activities designed to keep glucose levels in a safe, predefined target range to avoid both hyper- and hypoglycemia (by calculating and administering insulin doses matched to carbohydrate intake, balanced with physical activity and hormonal changes, among other factors affecting glycemic levels). This burden can cause considerable emotional distress and often negatively impact their quality of life (QoL) [1,2].

**Figure 1:**
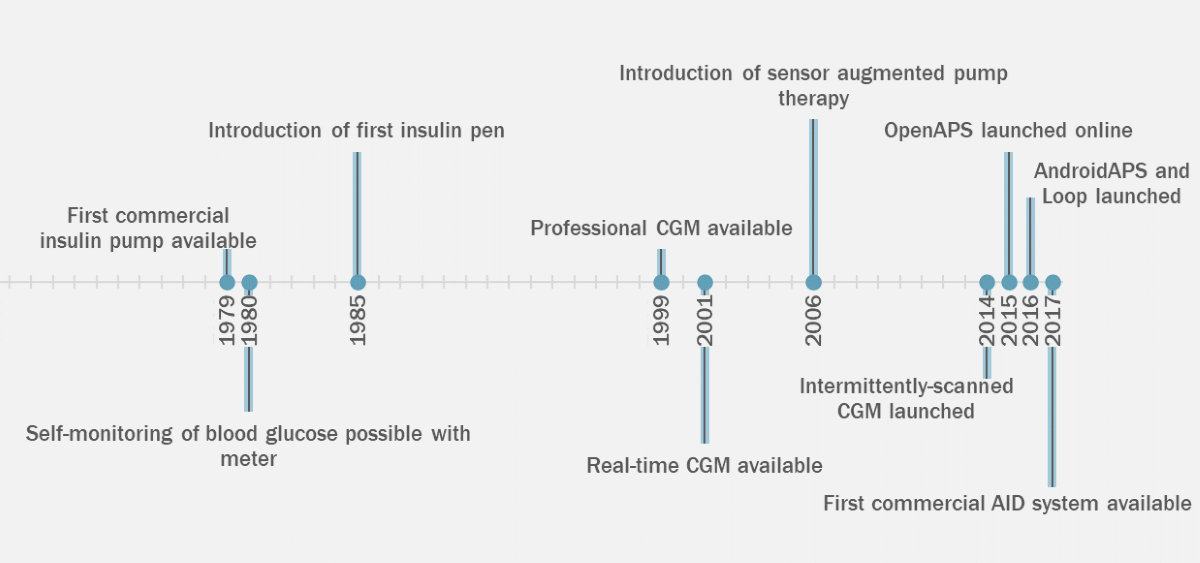
Timeline depicting the development of type 1 diabetes management and technology. This timeline is based on publications on insulin administration [3-5], glucose monitoring [6,7], intermittently scanned continuous glucose monitors (CGMs) [8], and open-source automated insulin delivery (AID) systems [9].

Advancements in diabetes treatments and technologies can assist in these two main tasks: (1) insulin administration and (2) glucose monitoring (Figure 1 [3-9]). Insulin can either be administered through multiple daily injections with a “pen injector” device or through an insulin pump, which is a wearable electronic device that pumps insulin through a thin plastic tube (cannula) inserted under the skin. Glucose levels can be monitored with a drop of blood (commonly collected by pricking a finger) or through a sensor inserted under the skin to assess glucose in the interstitial fluid using a continuous glucose monitor (CGM).

A recent review of randomized controlled trials (RCTs) and cohort studies investigating the impacts of diabetes technologies on psychosocial outcomes using validated person-reported outcome measures (PROMs) has shown that insulin pumps, CGMs, sensor-augmented pumps, and hybrid closed-loop technologies are often associated with considerable psychosocial benefits [10]. However, there appear to be fewer reported benefits in generic PROMs (eg, sleep quality) [10].

While insulin pumps and CGMs can improve glycemic and psychosocial outcomes [10], they do not automate the task of insulin delivery. Such automation would potentially reduce the burden and further improve outcomes for the individual. The most advanced diabetes technologies have the potential to do so, with automated insulin adjustments and dosing calculated through an algorithm linking a CGM to an insulin pump. These are known as automated insulin delivery (AID) systems. In recent years, both commercial and open-source AID systems have been developed [11]. Before commercial AID systems became available to the public, open-source AID systems were built by people with diabetes (and the parents of children with diabetes) using free-to-access algorithms (Figure 1) [12]. Several studies have demonstrated improved glycemic outcomes, consistently showing greater time-in-range and improved hemoglobin A_1c_ (HbA_1c_) [13].

In contrast to commercial systems, there has been relatively little research into the psychosocial outcomes of those using open-source AID systems. Early surveys showed benefits for sleep and QoL but were limited by the use of study-specific (unvalidated) survey questions [12,14,15]. Evidence of similar benefits has also been derived from qualitative studies, including content analysis of data shared through Twitter (subsequently rebranded X), which also demonstrated reduced diabetes burden [16,17]. A total of 2 small studies (N=35 and N=15) using validated PROMs have demonstrated benefits for diabetes distress [14] and fear of hypoglycemia [18]. Recently, a larger cohort study (N=239) found significant improvements, from baseline to 6 months of open-source AID use, in diabetes distress, sleep quality, fear of hypoglycemia, and hypoglycemia confidence [19]. To date, there has been no large-scale investigation of psychosocial outcomes using validated PROMs comparing those using and not using open-source AID systems. This has been considered a “missed opportunity” [20].

Thus, our aim was to compare the psychosocial outcomes of adults with type 1 diabetes using and not using open-source AID systems through the completion of validated PROMs in a multinational web-based survey.

## Methods

### Overview

The study design was cocreated by the “Outcomes of Patients’ Evidence with Novel, Do-it-Yourself Artificial Pancreas Technology Project Consortium” (OPEN) [21], which comprises an interdisciplinary team of clinicians, social scientists, data scientists, open-source AID developers, biomedical researchers, and public health researchers, many of them with lived experience in type 1 diabetes and in the use of open-source AID systems. This study was part of a larger, overall study conducted by OPEN. The overall survey was piloted with 8 volunteers from the diabetes online community. Minor edits were made based on their feedback.

We recruited participants through diabetes online community platforms (eg, Diabetes Daily), including Facebook groups supporting the open-source AID community (eg, Looped and AndroidAPS users), the OPEN international health care professional network [22], and the OPEN project Twitter and Facebook accounts, website, and newsletter. The survey was available in English only and open for data collection for 11 weeks (September-November 2020).

Participants were eligible for inclusion if they were adults (aged 18 years or older) with type 1 diabetes and attempted at least one PROM in the survey (eg, completed the first question). Adults with other types of diabetes were excluded from this analysis. Participants currently using an open-source AID system (a self-built automated insulin delivery system, either AndroidAPS, Loop, or OpenAPS) were included as the “user” group and self-reported their duration of use.

### Study Materials

The survey included 8 validated generic, diabetes-specific, and hypoglycemia-specific PROMs. Brief descriptions, scoring details, and interpretations are presented in Table S1 in Multimedia Appendix 1. Briefly, the psychosocial outcomes and measures included:

- General emotional well-being: 5-item World Health Organization Well-Being Index (WHO-5) [23]. This has been validated in adults with type 1 diabetes, has demonstrated reliability, and has been shown to have sensitivity and specificity for use as a screening tool for depressive symptoms among those with type 1 diabetes [24].
- Subjective sleep quality: Pittsburgh Sleep Quality Index (PSQI) [25]. The PSQI has demonstrated reliability and validity [26] and has previously been used in studies of adults with type 1 diabetes [27,28].
- Diabetes-specific quality of life: modified DAWN Impact of Diabetes Profile (DIDP) [29]. The 7-item DIDP has satisfactory psychometric properties and has been validated for use by adults with type 1 diabetes [29].
- Diabetes-specific positive well-being: 4-item subscale of the 28-item Well-being Questionnaire (W-BQ28) [30]. The W-BQ28 is a valid and reliable measure of generic and diabetes-specific well-being [31].
- Satisfaction with diabetes treatment: Diabetes Treatment Satisfaction Questionnaire (DTSQ) [32]. The DTSQ has satisfactory psychometric properties. It is reliable, has construct validity, demonstrates sensitivity to change, and has discriminatory power [32].
- Diabetes-specific distress: 20-item Problem Areas in Diabetes (PAID) scale [33]. The PAID is psychometrically robust for assessing diabetes distress [34].
- Fear of hypoglycemia: short form of the Hypoglycemia Fear Survey II (HFS-II SF) [35]. This matches the factor structure of the original instrument while maintaining reliability [36].
- Impact of the COVID-19 pandemic on QoL: COVID-19 Impact on Quality of Life scale [37]. Adapted from the validated 7-item DIDP [29].

Their selection was informed by our previous qualitative work [38] (summarized in Table S2 in Multimedia Appendix 1), previous surveys using unvalidated measures [12,15-17], and the lived experiences with open-source AID of the OPEN research team. At the time of measure selection, none of the studies using validated measures had been published. Where possible, we selected widely used measures to enable comparisons with published research. Overall participant burden and experience were also considerations, so we prioritized the selection of brief and, ideally, positively worded measures [39].

To characterize the study sample, we collected self-reported demographic data (ie, gender, age, country of origin, ethnicity, employment, income, and education level) and clinical data (ie, diabetes duration, most recent self-reported HbA_1c_, type of diabetes management, and presence of complications).

### Statistical Analysis

We performed all data processing and statistical analyses using SPSS Statistics (version 27; SPSS Inc). Missing data were dealt with according to guidance in the PROM scoring manuals (when available). When this guidance was not available, Cronbach α (and α if items were deleted) was used to guide whether missing data could be tolerated without loss of internal consistency reliability, enabling data to be imputed by using the mean response of the individual to that PROM. Total scores were calculated according to the developers’ guidance (Table S1 in Multimedia Appendix 1).

We used chi-square tests and independent groups 2-tailed *t* tests to assess differences in self-reported demographic and clinical characteristics and PROM scores by use or nonuse of open-source AID systems. For PROM scores, a Bonferroni-adjusted α level of *P*<.005 per test was accepted as significant, accounting for the number of *t* tests conducted. As assumptions of normality were not met, we also performed nonparametric tests (the Mann-Whitney *U* test). The significance remained unchanged; therefore, the results of the parametric analyses are reported alone. To adjust the analysis for potentially confounding variables, we used analysis of covariance tests, including as covariates all sociodemographic and clinical characteristics that showed differences by use of open-source AID.

### Ethical Considerations

Ethical approval was granted by the Life Sciences Human Research Ethics Committee, University College Dublin (LS-20-37) and the Ethics Committee of Charité–Universitätsmedizin Berlin (EA2/206/21). Potential participants had access to a participant information sheet and provided informed consent electronically (ticking a box) through REDCap (Research Electronic Data Capture; Vanderbilt University), a secure web application used to build and maintain web-based surveys and databases. The data have been deidentified. No financial compensation was provided to the participants.

## Results

**Figure 2:**
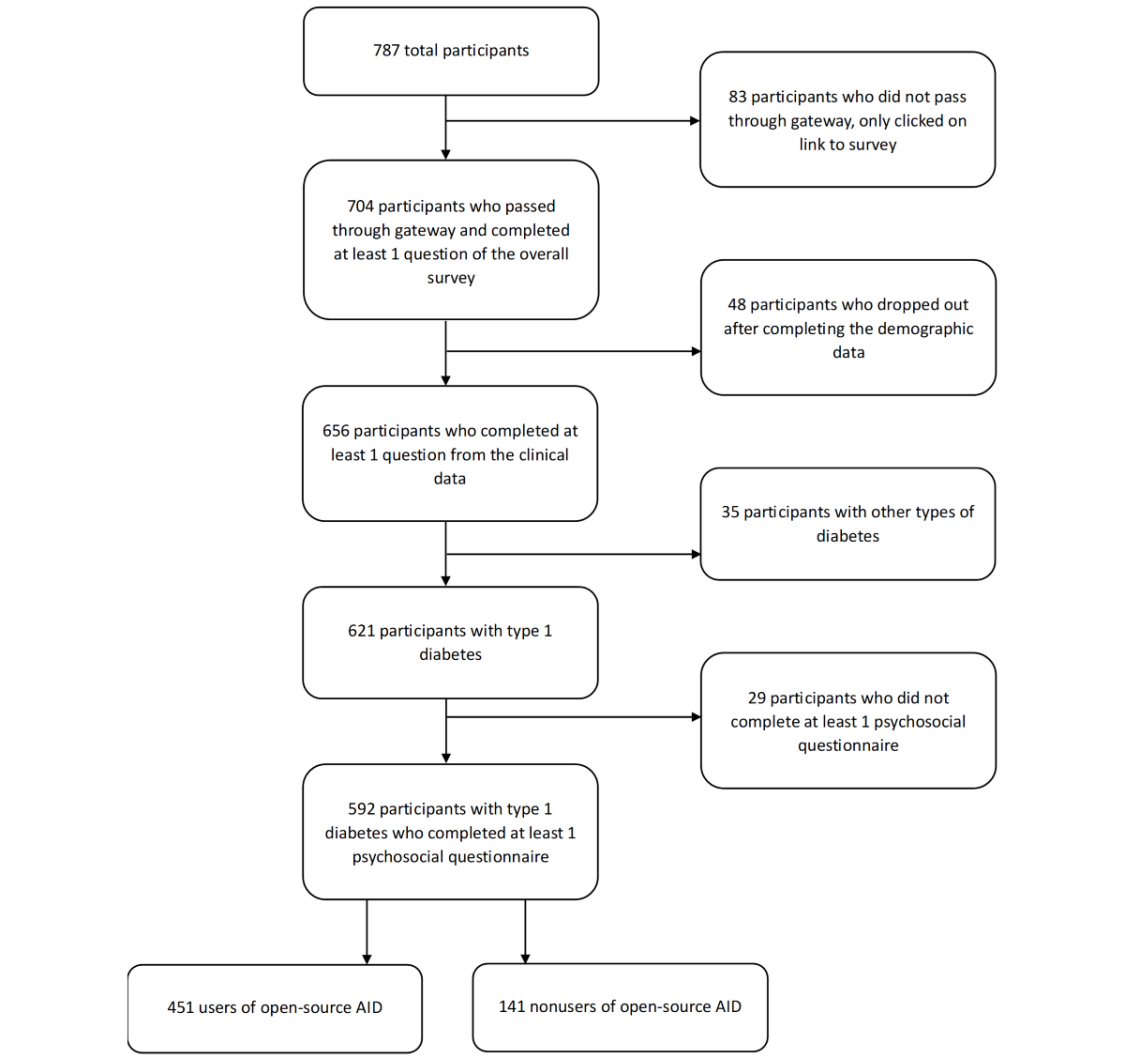
Diagram of participant inclusion and attrition. Adults with other types of diabetes (6 with type 2 diabetes, 21 with latent autoimmune diabetes, 6 with maturity-onset diabetes of the young, 1 with type 3c diabetes, and 1 participant with partial loss of pancreas) were excluded. AID: automated insulin delivery.

Figure 2 shows that, of the 787 people who responded to the survey advertisement, a total of 592 were eligible for inclusion in this analysis. Of these, a total of 451 individuals were using an open-source AID system (users) and 141 were not (nonusers).

Sociodemographic characteristics are reported in Table 1, and clinical characteristics are reported in Table 2. In this study, a total of 47 countries were represented, across all 6 of the World Health Organization regions. Participants using open-source AID systems (compared with nonusers) were more likely to be men and have higher income levels (Table 1).

Participants using open-source AID systems (compared with nonusers) had a longer duration of type 1 diabetes, lower self-reported HbA_1c_, and were less likely to have diabetes-related complications (Table 2).

Participants in the nonuser group managed their diabetes in various ways (Table 2). Most (128/141, 90.7%) were using some form of advanced diabetes technology, including commercial AID, sensor-augmented pump therapy, insulin pump therapy, and CGM. Some participants were using multiple methods simultaneously or switching between methods; a total of 32.6% (46/141) of participants were using a sensor-augmented pump or commercial AID system (with 1 participant reporting using both a sensor-augmented pump and a commercial AID system) in combination with other self-management methods. A total of 58.2% (82/141) of participants were not using a sensor-augmented pump or commercial-AID system but were using either a CGM, an insulin pump, or both in combination with other self-management methods. A minority (8/141, 5.7%) were using multiple daily injections and manual blood glucose checks alone, or in addition to inhaled insulin or sodium-glucose cotransporter-2 inhibitors. A further 3.5% (5/141) of participants did not report their methods of managing diabetes.

Table 3 shows between-group differences for PROM scale scores. Higher scores indicate greater emotional well-being, worse sleep quality, a greater negative impact of diabetes on QoL, greater diabetes-specific positive well-being, greater satisfaction with diabetes treatment, greater diabetes distress, greater fear of hypoglycemia, and a greater negative impact of the COVID-19 pandemic and restrictions on QoL. The number of participants who completed each questionnaire is reported in the “Completed questionnaires” column, with some questionnaires having lower completion rates due to attrition.

Compared with nonusers, adults with type 1 diabetes using open-source AID systems had greater general emotional well-being, subjective sleep quality, diabetes-specific QoL, diabetes-specific positive well-being, and diabetes treatment satisfaction (all *P*<.001). They also had lower diabetes distress, fear of hypoglycemia, and perceived less impact of the COVID-19 pandemic on their QoL than nonusers (all *P*<.001). All effect sizes were medium or large.

Where available, participants who fell above or below the cutoff scores for the PROMs were calculated. For the WHO-5, a total of 15.1% (68/451) of users and 35.5% (50/141) of nonusers reported a score <13, indicating likely depression. For the PSQI, a total of 32% (128/400) of users and 56.7% (63/111) of nonusers reported a score >5, indicating suboptimal sleep quality. For the PAID, a total of 14.1% (58/411) of users and 30.2% (38/126) of nonusers reported a score ≥40, indicating severe diabetes distress.

When adjusted for between-group differences in gender, region of origin, income level, diabetes duration, and most recent self-reported HbA_1c_, all between-group differences in PROM scores remained significant (*P*<.005), indicating that the differences between groups were not due to these sociodemographic or clinical characteristics.

**Table 1:** Participants’ self-reported demographic characteristics for users and nonusers of open-source automated insulin delivery (AID). Percentages may not add to 100 due to rounding.

			Users (n=451)		Nonusers (n=141)		*P* value
Gender (men)^a^, n (%)			262 (58.1)		50 (35.5)		<.001
Age (years)^a^, mean (SD)			43.0 (12.7)		40.4 (13.1)		.04
**Region of origin^a^, n (%)**							<.001
	African	4 (0.9)		0 (0)			
	Region of the Americas	67 (15)		38 (27.1)			
	South-East Asian	3 (0.7)		2 (1.4)			
	European	348 (77.8)		90 (64.3)			
	Eastern Mediterranean	1 (0.2)		4 (2.9)			
	Western Pacific	24 (5.4)		6 (4.3)			
**Ethnicity, n (%)**							.27
White			418 (92.7)		123 (87.2)		
**Education level, n (%)**							.41
	Incomplete high school	8 (1.8)		1 (0.7)			
	High school education	48 (10.6)		12 (8.5)			
	Undergraduate education	186 (41.2)		70 (49.6)			
	Postgraduate education	194 (43)		57 (40.4)			
**Occupational status, n (%)**							.68
	Employed (full or part-time)	351 (77.8)		103 (73)			
	Unemployed	15 (3.3)		7 (5)			
	Retired	35 (7.8)		11 (7.8)			
	Student or apprentice	33 (7.3)		13 (9.2)			
	None of the above or rather not say	17 (3.7)		9 (4.9)			
**Income level^a^ (US $), n (%)**							.002
	<49,999	96 (21.3)		50 (35.5)			
	50,000-99,999	140 (31)		41 (29.1)			
	100,000- 199,999	105 (23.3)		20 (14.2)			
	>200,000	27 (6)		4 (2.8)			

^a^*P*<.05 for between-group differences by use (or not) of open-source AID.

**Table 2:** Participants’ self-reported clinical characteristics for users and nonusers of open-source automated insulin delivery (AID). Percentages may not add to 100 due to rounding.

				Users (n=451)			Nonusers (n=141)			*P* value	
Diabetes duration (years)^a^, mean (SD)				26.7 (14.3)			21.6 (12.7)			<.001	
**Most recent self-reported HbA1c^a,b^ , mean (SD)**											<.001
	%		6.2 (0.9)			6.8 (1.0)					
	mmol/mol		44.7 (9.9)			50.5 (10.6)					
**Open-source AID type (users only)^c^, n (%)**											N/A^d^
	AndroidAPS		298 (66.1)			N/A					
	Loop		141 (31.3)			N/A					
	OpenAPS		36 (8)			N/A					
Open-source AID duration (years; users only), mean (SD)				1.77 (1.0)			N/A			N/A	
**Diabetes management (nonusers only)^c^, n (%)**											
	**Combined glucose monitor and insulin delivery**									N/A	
		Commercial AID system	N/A		23 (16.3)						
		Sensor-augmented pump therapy	N/A		24 (17)						
	**Glucose monitoring**									N/A	
		Continuous glucose monitor	N/A		77 (54.6)						
		Manual blood glucose checks	N/A		36 (25.5)						
	**Insulin delivery**									N/A	
		Insulin pump (not sensor augmented)	N/A		43 (30.5)						
		Multiple daily injections	N/A		46 (32.6)						
		Inhaled insulin	N/A		1 (0.7)						
	**Adjuvant diabetes medications**									N/A	
		SGLT2^e^ inhibitors	N/A		3 (2.1)						
Diabetes-related complications^f^; presence of at least 1^a^, n (%)				105 (25.9)			38 (31.4)			.004	

^a^*P*<.05 for between-group differences by use (or not) of open-source AID.

^b^HbA_1c_: hemoglobin A_1c_.

^c^Percentages do not add to 100% as some participants use multiple methods simultaneously or switch between methods.

^d^N/A: not applicable.

^e^SGLT2: sodium-glucose cotransporter-2.

^f^Complications count based on experiences of the following: retinopathy, kidney disease, peripheral neuropathy, digestive complications, foot complications, diabetes-related complications related to sexual health, and cardiovascular disease.

**Table 3:** Person-reported outcome measure (PROM) scores by use and nonuse of open-source automated insulin delivery.

Psychosocial outcome (PROM score range)			Users (n=451)			Nonusers (n=141)			Effect size		*P* value	
		Mean (SD)		Completed questionnaires, n	Mean (SD)		Completed questionnaires, n	Cohen *d*				
**Generic PROMs**												<.001
	General emotional well-being (WHO-5^a^; scored 0-25)	16.7 (4.5)		451	14.0 (4.8)		141	–0.6				
	Subjective sleep quality (PSQI^b^; scored 0-21)	4.9 (2.8)		400	6.4 (3.0)		111	0.5				
**Diabetes-specific PROMs**												<.001
	Diabetes-specific quality of life (modified DIDP^c^; scored 1-7)	4.3 (0.8)		426	4.8 (0.8)		129	0.6				
	Diabetes-specific positive well-being (4-item subscale of W-BQ28^d^; scored 0-12)	8.7 (2.3)		419	6.6 (2.8)		127	–0.9				
	Diabetes treatment satisfaction (DTSQ^e^; scored 0-36)	31.3 (3.7)		422	24.5 (6.8)		128	–1.5				
	Diabetes distress (PAID^f^; scored 0-100)	19.4 (15.8)		411	32.8 (20.8)		126	0.8				
**Hypoglycemia-specific PROMs**												<.001
	Fear of hypoglycemia (HFS-II SF^g^): behavior subscale (scored 0-20)	3.2 (3.1)		408	5.2 (4.3)		125	0.6				
	Fear of hypoglycemia (HFS-II SF): worry subscale (scored 0-24)	5.1 (4.1)		409	8.4 (5.0)		125	0.7				
**COVID-19–specific PROMs**												<.001
	Impact of COVID-19 pandemic on quality of life (COVID-19 Impact on Quality of Life Scale; scored 1-7)	4.6 (0.6)		394	4.9 (0.6)		113	0.5				

^a^WHO-5: 5-item World Health Organization Well-Being Index.

^b^PSQI: Pittsburgh Sleep Quality Index.

^c^DIDP: DAWN Impact of Diabetes Profile.

^d^W-BQ28: 28-item Well-Being Questionnaire.

^e^DTSQ: Diabetes Treatment Satisfaction Questionnaire.

^f^PAID: Problem Areas in Diabetes.

^g^HFS-II SF: short form of the Hypoglycemia Fear Survey II.

## Discussion

### Overview

This study is the first to use a comprehensive set of validated PROMs to examine the psychosocial outcomes of adults with type 1 diabetes using open-source AID systems, compared with those not using such systems. Those using open-source AID systems report significantly greater general emotional well-being, subjective sleep quality, diabetes-specific QoL, diabetes-specific positive well-being, and diabetes treatment satisfaction compared with nonusers. They also report significantly less diabetes distress and fear of hypoglycemia, as well as less negative impact of the COVID-19 pandemic and its restrictions on their QoL, compared with nonusers.

There were differences between those using and not using open-source AID systems in terms of gender, geographic region, income, diabetes duration, and most recent self-reported HbA_1c_. Importantly, the differences in PROM scores remained after adjusting for these differences, suggesting that these demographic and clinical differences do not explain the between-group differences in psychosocial outcomes. In the covariate analysis, the between-group difference in self-reported HbA_1c_ did not explain the differences in psychosocial outcomes. This suggests that these user-reported benefits are independent of glycemic outcomes. It is important to understand and appreciate the impact that diabetes technologies can have on the experiences of people with type 1 diabetes [3], not just their glucose levels. These include benefits such as a reduction in the effort required to manage their diabetes, improvements in sleep quality, and increased flexibility (Table S2 in Multimedia Appendix 1).

These results add to the existing qualitative and quantitative research (using unvalidated single items) supporting the beneficial psychosocial outcomes for users of open-source AID on sleep quality, diabetes distress, and QoL. This study is the first to use validated PROMS to assess general emotional well-being, diabetes-specific QoL, diabetes-specific positive well-being, and diabetes treatment satisfaction among open-source AID users and has demonstrated significant differences for all of these outcomes. This study corroborates the findings of 2 previous, but small (N≤35), open-source AID studies, which used validated PROMs to assess diabetes distress and fear of hypoglycemia, demonstrating reductions in both [14,18]. To date, 1 other study of open-source AID systems has used multiple validated PROMs in a large sample, showing significant improvements in sleep quality and significant reductions in diabetes distress and fear of hypoglycemia from baseline to 6 months following the initiation of an open-source AID system [19]. Those prospective findings are consistent with our cross-sectional analysis showing between-group differences. Furthermore, this study was multinational, across various health care systems and contexts, and found similar outcomes to their single-country (United States) study.

These findings need to be corroborated with other designs, such as RCTs, which are able to minimize and account for the demographic and clinical differences between participant groups, as well as psychosocial outcomes, before the adoption of open-source AID systems. Such designs will add to the evidence regarding the psychosocial impacts of open-source AID systems. The CREATE RCT is the first worldwide study to randomize adults (n=49) to open-source AID versus sensor-augmented pump therapy [40]. The findings demonstrate open-source AID to be safe, acceptable, and effective, with those using open-source AID spending over 3 hours more per day with glucose in the target range than those using sensor-augmented pump therapy [41,42]. The psychosocial results are forthcoming, based on the use of 4 PROMs: the EuroQol-5 Dimension [43], DTSQ, Hypoglycemia Fear Survey, and PSQI [40]. A preliminary conference presentation indicated no significant differences between groups in terms of QoL, diabetes treatment satisfaction, or fear of hypoglycemia (for the adult participants). However, interview data show participants reported improved psychosocial outcomes. The authors state that multiple factors may have impacted the PROM findings, including low numbers of participants and control arm participants having funded access to CGMs [44]. Comparatively, this study has shown significant differences between those using open-source AID and nonusers in QoL, diabetes treatment satisfaction, and fear of hypoglycemia, plus additional apparent benefits. This study involved larger samples than the CREATE trial; we had the power to demonstrate differences where they occurred. Alternatively, it may be due to differences in the experiences between the participants in our real-world study and the participants in the RCT. Our participants built their systems themselves and engaged with online peer support, which may have added benefits beyond supporting the “build.” Due to the design of the RCT, participants were provided with the AID system (they did not have to build it themselves) and received support from the researchers and other participants in the study (which the authors note was not comparable to the experience of peer support in the open-source community). It is likely that these differences may have led to different psychosocial experiences.

As highlighted by previous work, it is challenging to determine which PROMs to use among those using open-source AID systems [20]. There have also been concerns about whether existing measures, many of which were developed several decades ago, would be sensitive enough to the potential benefits of modern diabetes technologies [20]. In this study, we used 8 PROMs, all of which were sufficiently sensitive to demonstrate significant differences between open-source AID users and nonusers. The selection of these PROMs was informed by our earlier in-depth qualitative research [38] in addition to the lived experience of several of the authors. Several of the measures we selected have also been used in RCTs and cohort studies of advanced diabetes technologies, also demonstrating benefits [10]. This indicates the importance of being cognizant of what matters to adults with diabetes, how these issues are affected by living with diabetes and existing treatment regimens, and how new treatments and technologies can affect them [45]. Based on the results of this study, these PROMs could be used in future research on psychosocial outcomes among open-source AID users.

The strengths of this study include the use of validated PROMs and the careful selection of these PROMs. This study has been able to provide an understanding of the psychosocial outcomes of a subsection of the open-source AID community, including multinational reach and a large sample relative to other psychosocial studies of open-source AID.

However, there are also several limitations. Participants were recruited to this survey from several online communities, which may not be representative of those not involved in these communities. For example, both groups on average had relatively higher income levels and lower HbA_1c_ levels compared with the general population of adults with type 1 diabetes. The study was also subject to self-selection bias, and recruiting through the OPEN networks may have led to additional bias. Further, although this study has compared 2 groups, neither was allocated at random. Finally, this study is limited by the cross-sectional design, which means we are unable to draw conclusions about causality. Despite adjusting for many known confounders of psychosocial outcomes, there may be unknown reasons why the open-source AID group reports better psychosocial outcomes.

### Conclusion

In conclusion, this multinational study has responded to a call for research that uses validated measures to examine the psychosocial outcomes of adults with type 1 diabetes using open-source AID systems [13,20]. This real-world study shows that they report better psychosocial outcomes compared with nonusers, even when adjusted for sociodemographic and clinical characteristics. This study indicates that validated PROMs can quantify the sentiments previously expressed qualitatively (or using unvalidated single items) by the community [12,15-17]. Further research is needed to examine the reasons for these differences.

## Appendix

Multimedia Appendix 1 Supplementary tables.

## Abbreviations

AID: automated insulin delivery
CGM: continuous glucose monitor
DIDP: DAWN Impact of Diabetes Profile
DTSQ: Diabetes Treatment Satisfaction Questionnaire
HbA1c: hemoglobin A1c
HFS-II SF: short form of the Hypoglycemia Fear Survey II
OPEN: Outcomes of Patients’ Evidence with Novel, Do-it-Yourself Artificial Pancreas Technology Project Consortium
PAID: Problem Areas in Diabetes
PROM: person-reported outcome measure
PSQI: Pittsburgh Sleep Quality Index
QoL: quality of life
RCT: randomized controlled trial
W-BQ28: 28-item Well-being Questionnaire
WHO-5: 5-item World Health Organization Well-Being Index

## Data Availability

The data sets generated or analyzed during this study are available from the corresponding author on reasonable request.
