# Supplementary material for: Psychosocial Outcomes Among Users and Nonusers of Open-Source Automated Insulin Delivery Systems: Multinational Survey of Adults With Type 1 Diabetes
Source: J Med Internet Res. 2023 Dec 14;25:e44002. doi: 10.2196/44002 (PMC10755653; doi:10.2196/44002)
Supplement: Multimedia Appendix 1 [file jmir_v25i1e44002_app1.docx]

**Table S1.** Descriptions, scoring details, and interpretation for the psychosocial measures.

| Questionnaire name | Domain measured and number of items | Scoring and interpretation |
| --- | --- | --- |
| **Generic PROMs** | | |
| 5-item World Health Organisation Well-Being Index (WHO-5) | General emotional well-being, 5 items | Item scores: 0 - 5; 0 = At no time, 5 = All of the time. The raw score is calculated by summing responses to the five items. The raw score ranges from 0 to 25, representing worst possible to best possible emotional well-being. Scores <13 indicate likely depression. |
| Pittsburgh Sleep Quality Index (PSQI) | Subjective sleep quality, 19 items | 19 individual items are combined to form 7 category scores. Each of the 19 items are weighted on a 0 – 3 scale. A total score is calculated by summing the 7 category scores, giving a total score ranging from 0 to 21 (no difficulty to severe difficulty). Higher scores indicate worse sleep quality. |
| **Diabetes-specific PROMs** | | |
| DAWN Impact of Diabetes Profile (DIDP) - modified | Diabetes-specific quality of life, 7 items | Item scores: 1 – 7; 1 = a very positive impact, 4 = no impact; 7 = a very negative impact. A composite score is calculated by dividing the sum of the individual items by the number of complete responses (excluding N/A responses and missing responses). Higher scores indicate a greater negative impact of diabetes on QoL. |
| 4-item subscale of 28-item Well-being Questionnaire (4-item subscale of W-BQ28) | Diabetes-specific positive well-being, 4 items | Item scores: 0 – 3; 0 = not at all; 1 = sometimes; 2 = often; 3 = always. A total score is calculated by summing individual item scores, achieving a score between 0 – 12. Higher scores indicate the respondent feels a greater sense of satisfaction from managing their diabetes. |
| Diabetes Treatment Satisfaction Questionnaire (DTSQ) | Diabetes treatment satisfaction, 8 items | Item scores: 0 – 6; 6 = very satisfied; 0 = very dissatisfied  A total score is calculated by summing individual item scores for items 1 and 4-8. Total scores range between 0 and 36. Higher scores indicate greater satisfaction with the current diabetes treatment regimen.  Items 2 and 3 are treated individually and scored 0 – 6; with higher scores representing greater perceived frequency of high and low glucose levels. |
| Problem Areas in Diabetes (PAID) scale | Diabetes distress, 20 items | Item scores: 0 – 4; 0 = not a problem, 4 = serious problem  A total score is calculated by summing individual item scores (range 0 – 80) and multiplying by 1.25 to achieve a total score between 0 – 100. Higher scores indicate greater diabetes distress. Scores of >40 indicate severe diabetes distress. |
| **Hypoglycaemia-specific PROMs** | | |
| Hypoglycaemia Fear Survey II short-form (HFS-II SF) | Behaviours and worries related to fear of hypoglycaemia (low glucose), 11 items | Item scores: 0 – 4; 0 = never; 2 = sometimes; 4 = almost always  Two subscale scores are calculated by summing relevant item scores to form:  1) Behaviour subscale (5 items): ranging 0 to 20  2) Worry subscale (6 items): ranging 0 to 24  Higher scores indicate greater fear of hypoglycaemia. |
| **COVID-19-specific PROMs** | | |
| COVID-19 Impact on Quality of Life scale | Impact of COVID-19 pandemic on diabetes specific quality of life, 10 items | Item scores: 1 – 7; 1 = a very positive impact, 4 = no impact; 7 = a very negative impact.  A composite score is calculated by summing the individual items and dividing by the number of complete responses (excluding N/A responses and missing responses). Higher scores indicate greater negative impact of the COVID-19 pandemic and restrictions on QoL. |

**Table 2.** Examples of quotes from previous qualitative work that informed the selection of psychosocial outcomes and person-reported outcome measures.

| **Psychosocial outcome** | **Concepts** | **Quotes from Interviews** |
| --- | --- | --- |
| Sleep quality | Improvement to sleep quality | *“To be able to sleep without waking up in the middle of the night low, without waking up high. It’s just the quality of the sleep would have to be the greatest asset that I’ve found since I started looping.” (Participant ID 4, Female, using open-source AID for 1.2 years)* |
|  | Waking up feeling rested | *“I don’t wake up with hangovers anymore. So, I’m a non-drinker, so when I say a hangover, the feeling of a hangover.” (ID13, M, 0.6 years)* |
| Satisfaction with diabetes treatment | General satisfaction with treatment | *“I’m, overall, happy so far with what Loop’s doing.” (ID11, M, 0.2 years)* |
|  | Effectiveness | *“Rather than struggling with lows and highs, loop is helping me stay more in control.” (ID16, M, 0.8 years)* |
|  | Flexibility | *“Just being able to be flexible and variable, you know… it allows you to be more normal by a non-diabetic person’s perspective, I guess.” (ID4, F, 1.2 years)* |
|  | Convenience | *“Look, the main thing is that it’s a convenience factor. It makes the diabetes easier to manage.” (ID3, M, 0.6 years)* |
|  | Recommendation to others | *“I honestly do believe everyone could benefit from a DIY APS or some sort of closed loop system. It’s just, you can’t disregard the significant reduction of burden of disease on the individual with type 1 diabetes and their family.” (ID17, M, 0.3 years)* |
|  | Intention to continue with treatment | *[Interviewer] “Would you continue to use your DIY platform …?” [Participant] “100 percent.” (ID2, M, 2 years)* |
| Diabetes-specific emotional well-being  (both positive well-being and distress) | Feeling a sense of satisfaction from managing diabetes | *“From a mental health point of view, it does give you more sense of accomplishment. I made this. I built this…And I think you can’t beat that sense of ‘I did this, God I’m smart’.” (ID4, F, 1.2 years)* |
|  | Ability to cope with challenges | *“I have hope that this'll be something that I can manage” (ID19, F, 0.1 years)* |
|  | Reduction of worries about future complications | *“[Previously] I thought I might lose my toes and I might be in a wheelchair one day. [Now] I have more hope that I will be extending my quality of life as well as quantity so that I can hopefully just be a grandma’’ (ID19, F, 0.1 years)* |
|  | Acceptance of diabetes | *“It makes you feel at ease. You just feel more comfortable with your diabetes.” (ID6, M, 0.4 years)* |
|  | Reduction of constant effort to manage diabetes | *“Well, I feel like I'm not really managing it. It’s just doing it itself… A lot less effort on my part” (ID22, F, 1.2 years)* |
| Impact on quality-of-life domains | Impact on relationships | *“Three years ago, my wife, when I’m not at home, would be monitoring my blood glucose very cautiously or carefully…She doesn’t even think about looking for my BG results anymore because she knows that I’m nearly always sitting in that green range and she doesn’t have to worry about it.” (ID2, M, 2 years)* |
|  | Dietary freedom | *“I could if I wanted to have a meal that’s very high in carbs but the next day eat very little carbs” (ID4, F, 1.2 years)* |
| Fear of hypoglycaemia | *“Being able to go into a meeting and not [be] worried about going low because it would be mostly managed.” (ID1, F, 0.6 years)* | |
|  | *“I guess the fears of the hypos, that I could be more in target range without having to worry so much about the variability in a day.”* *(ID22, F, 1.2 years)* | |
